# Feasibility of Anesthesiologist-Performed Preoperative Echocardiography for the Prediction of Postinduction Hypotension: A Prospective Observational Study

**DOI:** 10.1155/2020/1375741

**Published:** 2020-10-20

**Authors:** Babar Fiza, Neal Duggal, Caitlin E. McMillan, Graciela Mentz, Michael D. Maile

**Affiliations:** ^1^Emory University School of Medicine, 1364 Clifton Road, Northeast Atlanta, GA 30322, USA; ^2^University of Michigan School of Medicine, Ann Arbor, MI, USA

## Abstract

**Purpose:**

To determine if left ventricular or inferior vena cava (IVC) measurements are easier to obtain on point-of-care ultrasound by anesthesiologists in preoperative patients, and to assess the relationship between preoperative cardiac dimensions and hypotension with the induction of general anesthesia.

**Methods:**

This prospective observational study was conducted at a large academic medical center. Sixty-three patients undergoing noncardiac surgeries under general anesthesia were enrolled. Ultrasound examinations were performed by anesthesiologists in the preoperative area. To ensure that hypotension represented both a relative and absolute decrease in blood pressure, both a mean arterial pressure (MAP) < 65 mmHg and a MAP decrease of >30% from preoperative value defined this outcome.

**Results:**

Left ventricular measurements were more likely to be acquired than IVC measurements (97% vs. 79%). Subjects without adequate images to assess IVC collapsibility tended to have a higher body mass index (33.6 ± 5.5 vs. 28.5 ± 4.5, *p*=0.001). While high left ventricular end-diastolic diameter values were associated with a decreased odds of MAP < 65 mmHg (OR: 0.24, 95% CI: 0.07–0.83, *p*=0.023) or a MAP decrease of >30% from baseline alone (OR: 0.25, 95% CI: 0.07–0.83, *p*=0.023), the primary endpoint of both relative and absolute hypotension was not associated with preoperative left ventricular dimensions.

**Conclusions:**

Preoperative cardiac ultrasound may be a more reliable way for anesthesiologists to assess patients' volume status compared to ultrasound of the IVC, particularly for patients with a higher body mass index.

## 1. Introduction

Decreased blood pressure can result in organ malperfusion and tissue damage. This occurs commonly with the induction of general anesthesia and initiation of positive-pressure ventilation [1]. Given its association with adverse events such as acute kidney injury and myocardial infarction, avoidance of perioperative hypotension is of paramount importance [2]. Finding new ways to identify patients at risk for perioperative hypotension will allow clinicians to appropriately adjust monitoring and treatment, which will reduce the occurrence of these adverse events.

Preoperative ultrasound examination performed by anesthesiologists is a valuable tool for identifying patients at risk for perioperative hypotension. Increased availability, superior image quality, and improved portability have resulted in an increased utilization of point-of-care (POC) ultrasonography in the perioperative setting. Zhang and Critchley demonstrated that respiratory variation in inferior vena cava (IVC) dimensions may predict postinduction hypotension in patients undergoing noncardiac surgery [3]. While IVC size and collapsibility index (IVC_CI_) are useful, an adequate view of the IVC could not be obtained in 13.5% of patients, and more than 20% of patients experiencing hypotension were missed. This percentage may increase when patients have increased abdominal adiposity. Furthermore, IVC collapsibility index measurements have low sensitivity in the prediction of hypotension associated with general anesthesia [4]. Therefore, other techniques are needed to improve our ability to identify at-risk patients.

Echocardiographic measurements obtained by POC exams are a promising strategy, and these measurements may provide a useful alternative to IVC measurements in patients with increased abdominal adiposity [5, 6]. Left ventricular (LV) dimensions can be easily obtained by anesthesiologists and provide information on systolic and diastolic function as well as preload [7, 8]. Reduced fractional shortening represents LV systolic dysfunction, decreased size may help detect hypovolemia, and thickened walls may identify individuals with diastolic dysfunction [9, 10]. With the increasing role of POC ultrasound in the care of a perioperative patient, evidence is needed to ensure the proper use of this technology.

With this in mind, this study was performed to accomplish two aims. First, we sought to compare the ability of anesthesiologists to obtain cardiac measurements compared to inferior vena cava images. Second, we assessed the feasibility of cardiac dimensions obtained immediately before surgery to identify patients at risk for postinduction hypotension.

We hypothesized that anesthesiologists would be able to obtain cardiac views more frequently than assessing the IVC_CI_ and that both would be associated with hypotension after the induction of general anesthesia.

## 2. Methods

### 2.1. Study Population

This study was approved by the institutional review board, and written informed consent was obtained from all subjects or a legal surrogate. Adult patients scheduled to undergo noncardiac surgery using general anesthesia were screened for inclusion. Patients were excluded if they had known reduced left ventricular ejection fraction, were already admitted to the hospital, used mechanical ventilation at home, were prescribed systemic steroids, continued medications that inhibit the renin-angiotensin-aldosterone system, had preexisting wall motion abnormalities, underwent awake fiber-optic intubation, were dialysis dependent, or did not provide consent. The anesthesiologist performing the echocardiographic exam did not participate in the anesthetic management of the examined subject in order to avoid having the ultrasonographic findings from altering the induction strategy. Baseline patient characteristics were obtained from the electronic medical record.

A data analysis and statistical plan was written and filed with the institutional anesthesiology clinical research committee before data were accessed. The average and standard deviation of adult male left ventricular end-diastolic diameter (LVIDd) is 50.2 ± 4.1 mm. Assuming a 40% incidence of hypotension, examining 50 patients would allow us to detect a 3.3 mm difference in means between cases and controls with 80% power and an alpha of 0.05. An additional 25% of subjects were screened to account for any potential deviation from the protocol, and thus, a total of 67 patients were enrolled in the study.

### 2.2. Transthoracic Echocardiography

Transthoracic echocardiography examinations were performed by two anesthesiologists (Babar Fiza and Neal Duggal) who routinely use echocardiography in their clinical practice. These anesthesiologists have an extensive background in the practice of advanced perioperative and critical care echocardiography. Both clinicians had at least 4 years of experience in the field, and each had personally performed more than 150 echocardiographic examinations prior to the initiation of the study. The Philips Sparq (Philips Ultrasound, Bothell, WA, USA) and SonoSite X-Porte (FujiFilm SonoSite, Bothell, WA, USA) ultrasound machines were used to conduct the exams. Findings from each examination were reviewed by the performing anesthesiologist and confirmed by the second anesthesiologist who was blinded to the findings of the first examiner. The order of the ultrasound examination was kept uniform. The sequence of the examination included parasternal long-axis view followed by the parasternal short-axis view with the subcostal IVC as the final obtained view.

**Figure 1:**
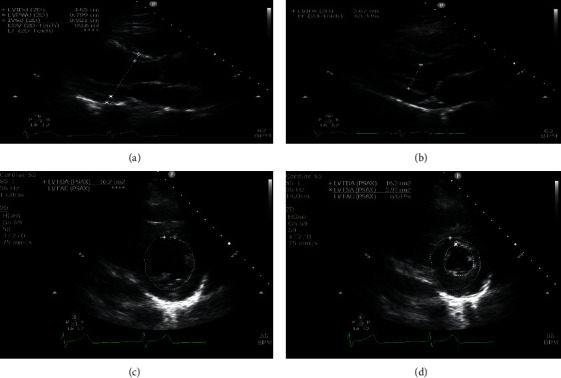
(a) Measurements of left ventricular chamber size and wall thickness dimensions in the parasternal long-axis view during diastole; (b) measurement of the left ventricular chamber size dimension in the parasternal long-axis view during systole. The left ventricular areas are measured at the midpapillary level of the left parasternal short-axis view during both diastole and systole; (c) left ventricular end-diastolic area; (d) left ventricular end-systolic area. LVIDd: left ventricular internal diameter diastole; LVPWd: left ventricular posterior wall diastole; IVSd: interventricular septum diastole; EDV: end-diastolic volume; EF: ejection fraction; LVIDs: left ventricular internal diameter systole; LVEDA: left ventricular end-diastolic area; LVFAC: left ventricular fractional area of change; LVESA: left ventricular end-systolic area.

The TTE examination consisted of parasternal (3^rd^ to 4^th^ intercostal space) and subcostal imaging in the supine position. If necessary, for image optimization, left lateral decubitus positioning was utilized. The left ventricular wall thickness and diastolic chamber dimensions were obtained from the parasternal long-axis view. In accordance with the American Society of Echocardiography guidelines, left ventricular wall thickness and diastolic chamber dimensions were made at end-diastole, defined as the first video frame immediately after mitral valve (MV) closure and/or at the peak of the R wave on the electrocardiogram. Measurements were obtained at the level just below the MV leaflet tips and interventricular septum and LV posterior wall was measured at the same time and level as the LV end-diastolic dimension (Figure 1(a)). The LV end-systolic dimension was obtained at the smallest cavity dimension, which coincided with the frame preceding the diastolic opening of the mitral valve and/or the end of the T wave on the electrocardiogram (Figure 1(b)).

LV end-diastolic area (LVEDA) and LV end-systolic area (LVESA) were obtained from parasternal short-axis view by tracing the endocardial border by planimetry, including the papillary muscles, in the end-diastolic and end-systolic frames, respectively (Figures 1(c) and 1(d)). The LVEDA was indexed to patient's body surface area to obtain left ventricular end-diastolic area index (LVEDAi). All obtained measurements are summarized in Table 1. All measurements were performed after the acquisition of the images, and for each patient, the best quality images were selected for analysis.

### 2.3. IVC Ultrasound

**Figure 2:**
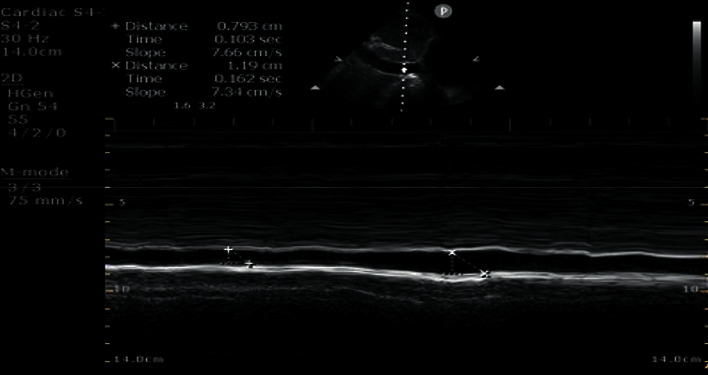
The maximum inferior vena cava diameter (dIVC_max_) was measured as the maximum anterior-posterior dimension at end-expiration using the leading-edge technique (inner edge to inner edge of the vessel wall). The minimum inferior vena cava diameter (dIVC_min_) was measured at end-inspiration. The collapsibility index was calculated using the built-in software available on the ultrasound systems. IVC: inferior vena cava; dIVC_max_: maximum IVC diameter; dIVC_min_: minimum IVC diameter.

The IVC was imaged in a longitudinal plane via the subcostal window. The IVC was differentiated from the aorta by visualizing its entrance into the right atrium and the hepatic vein drainage into the IVC. Respiratory variations in the IVC diameter were assessed using motion mode imaging. The maximum and minimum diameter measurements of the IVC were performed around 2 cm caudal to the hepatic vein-IVC junction. The maximum IVC diameter (dIVC_max_) was measured as the maximum anterior-posterior dimension at end-expiration using the leading-edge technique (inner edge to inner edge of the vessel wall). The minimum IVC diameter (dIVC_min_) was measured at end-inspiration. For each patient, the best quality image was chosen for analysis. The collapsibility index was calculated using the built-in software available on the ultrasound systems (Figure 2). The IVC_CI_ was calculated as IVC_CI_ = (dIVC_max_ − dIVC_min_)/dIVC_max_ and was expressed as a percentage.

### 2.4. Perioperative Anesthesia Practice

The preoperative and intraoperative management of the patient were left at the discretion of the anesthesia team. Clinicians were not informed of the echocardiographic measurements nor the specific goals of the study. Members of the treating anesthesia team were not allowed to be present at the bedside for the entire duration of the examination. If an unexpected and clinically relevant finding was appreciated during the exam, the intraoperative team was notified, and the subject was excluded from the analysis. The choice of noninvasive blood pressure or invasive blood pressure monitoring was determined by the treating anesthesiologist. Noninvasive blood pressure measurements were obtained at three-minute intervals per the institutional guidelines.

All patients were induced with propofol with an average total dose of 160 mg with a standard deviation of 41.5 mg. The average weight-based dose of propofol for the cohort was 1.86 mg/kg with a standard deviation of 0.34 mg/kg. More than 90% of the individuals (59/63) received fentanyl at the time of induction. The average total dose of administered fentanyl at the time of induction was 125 mcg. 46 patients received preoperative midazolam for anxiolysis with the dose range of 1–2 mg. All patients received controlled mechanical ventilation after induction of anesthesia with a minimum positive end‐expiratory pressure (PEEP) of 5 cm H_2_O.

### 2.5. Outcomes

The primary outcome was both a mean arterial pressure (MAP) < 65 mmHg and a decrease in MAP of at least 30% from the subject's preoperative value prior to the surgical incision. This a priori definition was selected to identify the presence of both absolute and relative hypotension. Secondary outcomes included the incidence of a MAP < 65 mmHg, the incidence of a MAP decrease of ≥ 30% from baseline, the total amount of time needed to complete the exam, and the frequency that adequate images were not obtainable.

### 2.6. Statistical Analysis

Exploratory data analysis techniques such as frequencies, means, medians, standard deviation, minimum and maximum, histograms, box plots, and QQ plots were used to assess the distribution of outcome measures as well as relevant predictors. Extreme values were identified and removed if they were determined to be influential. Counts and percentages were used to summarize categorical variables. The average ± the standard deviation was used to summarize continuous variables.

Echocardiographic data were analyzed as continuous variables and summarized as either the mean and standard deviation or median and interquartile range as appropriate. Continuous variables were summarized using means and standard deviations and categorical ones using counts and percentages. Potential selection bias was tested by comparing those that had IVC measurements to those that did not. Similarly, we compared those that had cardiac measurements to those that did not.

To test our research question related to the performance of echocardiographic variables, we used a sequential logistic regression approach where each relevant predictor was considered in a “one-at-a-time” approach, followed by a second model where gender and the interaction of gender and the predictor were added. Goodness of fit of the models was determined using AIC, SC, and −2loglikehood.

## 3. Results

A total of 67 adult patients were enrolled. Two individuals were excluded because their anesthetic plan was changed from general anesthesia to a sedation anesthetic, one subject was excluded as consent was obtained from the patient, but the patient was transferred to the operating room prior to the performance of the exam, and one subject was excluded when an unexpectedly reduced left ventricular function was appreciated during the exam.

Final data analysis included 63 patients, 60.3% men and 39.7% women, with a median age of 64.1 years and an average body mass index of 29.6. Of the 63 participants, 33 were classified as ASA II and 30 as ASA III based on the American Society of Anesthesiologists physical status classification system. A preoperative diagnosis of hypertension was present in about half of the study population (*n* = 32, 50.4%).  Table 2 summarizes the characteristics of the study population and compares those in which IVC measurements were and were not possible. IVC measurements were obtained in 79% of individuals (*n* = 50). In contrast, 97% had adequate images to measure left ventricular dimensions (*n* = 61). Only anthropometric variables differed between those with and without images that were adequate for assessing the IVC collapsibility index. For example, those in whom the IVC could not be assessed had a higher body mass index (BMI) (33.6 ± 5.5 vs. 28.5 ± 4.5, *p*=0.001).

The average time spent performing the echocardiography exam was 485.8 seconds (∼8 minutes). The left ventricular area was measurable in 87.3% of subjects. The average LVIDd of our study population was 4.4 ± 0.5 cm, and the average LVEDAi was 7.5 ± 1.9 cm^2^/m^2^. Two individuals had a virtual IVC (the IVC collapsed completely with inspiration). The average IVC_CI_ was 36.0 ± 21.1% (Table 1).

Regarding the incidence of induction-related hypotension, 44 subjects (70%) had a MAP < 65 mmHg during induction of anesthesia while 43 (68%) had a MAP recorded that was decreased > 30% from their preoperative value. Thirty-one individuals (49%) experienced the primary outcome of both a MAP < 65 mmHg and a decrease of >30% from the preoperative value (Table 2).

Unadjusted logistic regression models of each of the three outcomes showed similar patterns of association of both left ventricular dimensions and IVC variability with each individual outcome (Table 3). However, the primary outcome did not reach statistical significance. Increased LVIDd was associated with a decreased odds of a MAP < 65 mmHg (OR: 0.24, 95% CI: 0.07–0.83, *P*=0.023) and a 30% decrease in MAP from their preoperative value (OR: 0.25, 95% CI: 0.07–0.83, *P*=0.023). The composite outcome demonstrated a similar relationship despite not reaching statistical significance (OR: 0.40, 95% CI: 0.14–1.14, *P*=0.086). A similar relationship existed for LVEDA, although the magnitude of the effect was not as great. Higher values were associated with less hypotension defined as a MAP < 65 mmHg (OR: 0.86, 95% CI: 0.73–1.00, *P*=0.49) and a decrease of >30% from baseline (OR: 0.86, 95% CI: 0.73–1.00, *P*=0.049). Again, the composite outcome had a similar, although not statically significant, relationship (OR: 0.89, 95% CI: 0.78–1.02, *P*=0.091). Regarding IVC_CI_, higher values were associated with a small increase in the incidence of MAP < 65 mmHg (OR: 1.05, 95% CI: 1.00–1.10, *P*=0.039) and the incidence of a MAP decrease of > 30% from baseline (OR: 1.05, 95% CI: 1.00–1.10, *P*=0.032). Similar patterns were observed for the composite outcome, although they did not reach statistical significance (OR: 1.01, 95% CI: 0.98–1.04, *P*=0.671).

Ultrasound measurements that were associated with induction-related hypotension were entered into a multivariable regression that included both gender and the interaction of the predictor with gender. The odds ratio for LVIDd was 0.30, although this did not reach statistical significance (95% CI: 0.10–1.3, *p*=0.097). No other measurements remained associated with any of our criteria for hypotension with induction of general anesthesia after adjusting for gender (Table 4).

## 4. Discussion

This study supports the feasibility of anesthesiologist-performed preoperative echocardiography for the prediction of postinduction hypotension. These measurements were able to be made in a greater proportion of patients than measurement of IVC collapsibility, which is the most well-studied ultrasound measurement for assessing volume status. While we did not find an association between left ventricular dimension and our primary outcome, this was likely due to the small sample size of this pilot study. Given the exploratory nature of this study, we also examined the relationship between each component of the primary outcome, which used both an absolute and relative MAP for defining postinduction hypotension. Individually, either MAP < 65 mmHg or a decrease in MAP > 30% from the baseline value did have an association with static measures of left ventricular size (LVIDd and LVEDA).

Crucial for future studies investigating the optimal use of perioperative POC ultrasound, we also demonstrated that measurements of cardiac dimensions were more frequently available, particularly in patients with a higher BMI. This is likely due to abdominal adiposity increasing the difficulty of IVC assessment in patients with a higher BMI. A similar pattern did not exist for left ventricular cardiac dimensions and supports developing strategies for using echocardiographic measures to identify patients at increased risk for anesthesia complications especially in populations with a high incidence of obesity.

We also confirmed a previously described relationship between IVC_CI_ and hypotension with the induction of anesthesia. This relationship was found despite not controlling for any aspects of anesthesia management, which adds to the robustness of this finding. Additionally, etomidate was used as the agent of induction in the study by Zhang and colleagues, whereas our patients received propofol for induction [3], thus extending these findings to other types of clinical practice.

The association between the incidence of hypotension and specific ventricular dimensions or IVC values did not exist when hypotension was defined as a combination of both relative and absolute thresholds. We selected this definition as our a priori primary outcome to ensure that all induction-related hypotension was clinically significant. This characterization was also selected to account for the large variability in how intraoperative hypotension is defined in the literature. For instance, Bijker and colleagues noted 140 definitions of hypotension in 130 articles and observed that the incidence of intraoperative hypotension ranged from 5 to 99% depending on the numerous threshold values [11]. While an exact definition of intraoperative hypotension remains unclear, both absolute thresholds of MAP < 65 mmHg and a relative threshold of MAP < 30% from baseline are associated with organ injury and death [12]. A 30% reduction in MAP from baseline has been associated with postoperative stroke in patients undergoing noncardiac or nonneurosurgical procedures [13]. Additionally, intraoperative MAP < 65 before surgical incision has been strongly associated with both myocardial and kidney injury [2, 14, 15].

Given that the individual components of our composite outcome were associated with the echocardiographic measurements, this may suggest that TTE is more sensitive for detecting less severe hypotension, although this will have to be examined by future studies. This is of importance given the findings of associations between brief periods of even mild hypotension with myocardial injury, kidney injury, and mortality [2, 16, 17].

Point-of-care ultrasonography is rapidly being adopted by perioperative physicians. In the United States, comprehensive echocardiograms are routinely performed by the ultrasound technicians and interpreted later by the cardiologists, thus precluding its routine use in the fast-paced perioperative period. POC echocardiography on the other hand is a limited bedside investigation that is performed and interpreted by a physician at the bedside. This allows the anesthesiologist to examine cardiac status noninvasively in real time, reach rapid diagnosis, and perform repeat exams to assess response to the interventions. While this technology is being increasingly used in the perioperative period, limited data exist regarding the role of anesthesiologist-performed point-of-care studies on the intraoperative care and their association with perioperative outcomes. When conducted on patients undergoing noncardiac surgery, POC echocardiographic findings were associated with both changes in patient care and decreased postoperative adverse events [18–20]. Currently, more studies are needed to determine the role of POC echocardiographic measurements on identifying patients at risk for perioperative hypotension.

To our knowledge, this is the first study examining the usability of anesthesiologist-performed preoperative transthoracic echocardiography to identify echocardiographic parameters that are associated with postinduction hypotension. Left ventricular end-diastolic area and diameter measurements have been proposed to diagnose hypovolemia intraoperatively on transesophageal echocardiography (TEE), and changes in LVEDA have been noted to result in correlating changes in stroke volume and cardiac output [21]. The literature surrounding LVEDA measurements and their correlation with hypovolemia have been limited to intraoperative TEE measurements in patients undergoing cardiac surgeries. A study of 20 patients undergoing elective coronary artery bypass graft procedures found a decrease in LVEDA from baseline of 12.5 cm^2^ to 10.6 cm^2^ at the time of sternal closure. The decreased LVEDA was proposed to result from inadequate preload after sternal closure [22]. In a study involving 35 anesthetized cardiac surgical patients, a significant decrease in LVEDA was detected in response to 2.5% estimated blood volume deficit [23]. In pig models undergoing general anesthesia, LVEDA was noted to decrease from 13.8 cm^2^ to 5.1 cm^2^ during hemorrhage involving withdrawal of blood volume of 35 ml/kg [24]. In cardiac surgical patients, on TEE examination, mean EDA for patients with normal left ventricular function is noted to be 10.66 cm^2^ and 5.6 cm^2^/m^2^ when indexed to body surface area, although 85% of the study population consisted of male patients [25].

Over time, these findings have been applied to POC cardiac ultrasonography despite lack of validation studies, and an LVEDA of less than 10 cm^2^ or an LVEDA index of less than 5.5 cm^2^/m^2^ is proposed to indicate hypovolemia [26–28]. In the absence of papillary muscle inclusion in the LVEDA measurements, an LVEDA < 8 cm^2^ has been proposed to be consistent with hypovolemia [29]. However, normal values for ventricular dimensions may vary amongst patients depending on baseline cardiac anatomy and physiology [30]. In our work, we did not find any statistical significance between preoperative TTE values of LVEDA < 10 cm^2^ or LVEDAi < 5.5 cm^2^/m^2^ with postinduction hypotension.

Currently, limited data exist in the optimal workflow integration of POC ultrasound during the perioperative period. Perceived time constraints pose a significant challenge in the routine incorporation of POC ultrasound in the practice of anesthesiology. Our study establishes that POC cardiac ultrasound can be performed in a time-efficient manner during a routine clinical setting. The average time spent performing the echocardiography exam during our study was about eight minutes. The fastest exams in the study were performed within two minutes. The average timeframe was most likely lengthened by the examiners obtaining multiple images and attempts to obtain the perfect images for interpretation. In addition to the feasibility of anesthesiologist-performed TTE, we found that the echocardiographic images are easier to obtain than IVC measurements in certain populations and that several simple measurements appear to have a relationship with induction-related hypotension. Our findings can be used to power future studies. These findings also support the use of anesthesiologist-performed surface echocardiography especially as TTE has the advantage in screening for cardiac pathologies relevant to the practice of anesthesiology. This is highlighted by the fact that one of the enrolled patients was excluded from our analysis due to an unexpected preoperative finding of left ventricular systolic dysfunction. The abnormal cardiac findings were relayed to the intraoperative team in this case which resulted in an alteration of the anesthetic plan.

Several limitations should be considered when interpreting these findings. First, by not controlling for the medications used for the induction of anesthesia, practices such as administering vasoconstrictors to patients felt to be at high risk for hypotension may have altered relationships in an unpredictable way. However, by not sharing echocardiographic measures with clinicians and studying these predictors in the setting of routine clinical practice increase the external validity of the results. Second, echocardiographic exams were performed by anesthesiologists with experience in transthoracic echocardiography. It is possible that the accuracy of the measurements could be improved if performed by level three certified cardiologists or experienced sonographers. However, again, this would not be consistent with routine clinical practice. Finally, associations that were found between echocardiographic measurements and hypotension did not persist after adjusting for patient factors. While these relationships may persist with a larger sample size, this may also reflect the relationship that exists between echocardiographic measurements and patient factors.

In conclusion, we found that several echocardiographic measurements are associated with certain definitions of hypotension with the induction of anesthesia. We also demonstrated that cardiac dimensions are able to be measured more frequently than IVC dimensions, particularly in patients with a higher body surface area.

## Figures and Tables

**Table 1:** Echocardiographic measurements (*n* = 63).

Variable	*N*	Mean (SD)	(Min–max)
LVIDd (cm)	61	4.4 (0.5)	(3.1–5.6)
LVIDs (cm)	56	2.8 (0.5)	(1.7–4.0)
PWT (cm)	62	0.9 (0.2)	(0.6–1.6)
IVS (cm)	62	0.9 (0.2)	(0.1–1.4)
LVEDA (cm^2^)	55	14.9 (4.3)	(6.2–24.7)
LVESA (cm^2^)	55	5.3 (2.0)	(1.5–11.5)
LVEDA index (cm^2^/m^2^)	55	7.5 (1.9)	(3.0–12.1)
IVC_max_ (cm)	50	1.8 (0.4)	(1.0–2.6)
IVC_min_ (cm)	50	1.2 (0.5)	(0.4–2.4)
Collapsibility index (%)	50	36.0 (21.1)	(6.4–100.0)
Time to complete TTE exam (seconds)	63	483.0 (215.5)	(120.0–1175.0)

IVS, interventricular septum; IVC_max_, maximal diameter of the inferior vena cava; IVC_min_, minimal diameter of the inferior vena cava; LVIDD, left ventricle internal diameter at end of diastole; LVIDS, left ventricular internal diameter at end of systole; PWT, posterior wall thickness.

**Table 2:** Patient characteristics for all subjects and by the ability to obtain IVC measurements.

	All available data (*n* = 63)			(A) IVC measurements available (*n* = 48)			(B) IVC measurements not available (*n* = 15)			Comparison (A) vs. (B)
	*N*	%	Mean (SD)	*N*	%	Mean (SD)	*N*	%	Mean (SD)	*p* value
*Demographics*										
Age	63	—	64.1 (10.9)	48	—	62.8 (12.0)	15	—	68.1 (5.1)	0.098
Gender										
Female	25	39.7	—	22	45.8	—	3	20.0	—	0.074
Male	38	60.3	—	26	54.2	—	12	80.0	—	—
Height	63	—	171.0 (9.7)	48	—	170.5 (10.3)	15	—	172.5 (7.5)	0.485
Weight	63	—	87.3 (15.8)	48	—	83.5 (14.0)	15	—	99.4 (15.6)	<0.001
BMI	63	—	29.6 (5.2)	48	—	28.4 (4.5)	15	—	33.6 (5.5)	0.001
BSA (m^2^)	63	—	2.0 (0.5)	48	—	1.9 (0.2)	15	—	2.1 (0.2)	0.004
ASA physical status										
2	33	52.4	—	25	52.1	—	8	53.3	—	0.933
3	30	47.6	—	23	47.9	—	7	46.7	—	

*Health characteristics*										
Hypertension	32	50.4	—	23	47.9	—	9	60.0	—	0.414
CVD	2	3.2	—	2	4.2	—	0	—	—	—
CAD	4	6.3	—	4	8.3	—	0	—	—	—
Preoperative MAP	63	—	99.7 (13.6)	48	—	99.3 (13.4)	15	—	101 (14.4)	0.687

*Outcomes*										
MAP < 65 mmHg	44	69.8	—	32	66.7	—	12	80.0	—	0.520
MAP decrease > 30% preoperative value	43	68.3	—	31	64.6	—	12	80.0	—	0.350
MAP < 65 mmHg and decrease of > 30%	31	49.2	—	24	50.0	—	7	46.7	—	0.822

ASA, American Society of Anesthesiologists; BMI, body mass index; CVD, cardiovascular disease; IVC, inferior vena cava; MAP, mean arterial pressure (mmHg).

**Table 3:** Unadjusted logistic regression models including one predictor at a time (*n* = 63).

Predictors	Both			MAP < 65 mmHg			MAP decrease >30% of baseline		
	OR	95% CI	*p* value	OR	95% CI	*p* value	OR	95% CI	*p* value
LVIDd (cm)	0.40	(0.14,1.14)	0.086	0.24	(0.07,0.83)	0.023	0.25	(0.07,0.83)	0.023
LVIDs (cm)	0.84	(0.29,2.41)	0.742	0.76	(0.25,2.33)	0.635	0.79	(0.26,2.36)	0.666
PWT (cm)	0.50	(0.04, 6.50)	0.597	0.20	(0.01, 3.00)	0.242	0.20	(0.01, 2.99)	0.243
IVS (cm)	0.09	(0.01, 1.71)	0.109	0.05	(0.00, 1.51)	0.086	0.06	(0.00, 1.62)	0.095
LVEDA (cm^2^)	0.89	(0.78, 1.02)	0.091	0.86	(0.73, 1.00)	0.049	0.86	(0.73, 1.00)	0.049
LVESA (cm^2^)	0.89	(0.68, 1.18)	0.420	0.77	(0.57, 1.05)	0.094	0.77	(0.57, 1.05)	0.094
LVEDAi	0.82	(0.62, 1.10)	0.185	0.76	(0.55, 1.07)	0.116	0.76	(0.55, 1.07)	0.116
Collapsibility index (%)	1.00	(0.98, 1.04)	0.671	1.05	(1.00, 1.10)	0.039	1.05	(1.00, 1.10)	0.032
LVEDA < 10 cm^2^	2.2	(0.4, 11.99)	0.3621	7.616	(0.34, 172.23)	0.202	7.616	(0.34, 172.23)	0.202
LVEDAi < 5.5 cm^2^/m^2^	1.507	(0.33, 6.98)	0.600	8.91	(0.41, 194.68)	0.165	8.906	(0.41, 194.68)	0.165

IVS: interventricular septum; LVIDd: left ventricular internal diameter diastole; LVIDs: left ventricular internal diameter systole; LVEDA: left ventricular end-diastolic area; LVESA: left ventricular end-systolic area; LVEDAi: left ventricular end-diastolic area index; PWT: posterior wall thickness.

**Table 4:** Adjusted logistic regression models including one predictor at a time (*n* = 63).

	Both			MAP < 65 mmHg			MAP decrease >30% of baseline		
Predictors	OR	95% CI	*p* value	OR	95% CI	*p* value	OR	95% CI	*p* value
LVIDd (cm)	0.5	(0.1–2.2)	0.389	0.3	(0.1–1.3)	0.097	0.3	(0.1–1.3)	0.11
LVEDA (cm^2^)	0.9	(0.8–1.1)	0.44	0.9	(0.7–1.1)	0.197	0.9	(0.7–1.1)	0.197
IVC_CI_ (%)	0.99	(0.95,1.04)	0.654	1.03	(0.98,1.08)	0.276	1.03	(0.98,1.08)	0.253

Models adjust for gender (ref = male), predictor, and gender ^*∗*^ predictor. IVC_CI_: inferior vena cava collapsibility index; LVIDd: left ventricular internal diameter diastole; LVEDA: left ventricular end-diastolic area.

## Data Availability

The datasets generated and analyzed during the current study are available from the corresponding author upon request.

## Abbreviations

MAP: Mean arterial pressure
POC: Point-of-care
IVC: Inferior vena cava
IVC_CI_: IVC collapsibility index
LV: Left ventricle
LVIDd: Left ventricular end-diastolic diameter
MV: Mitral valve
LVEDA: Left ventricular end-diastolic area
LVESA: Left ventricular end-systolic area
LVEDAi: Left ventricular end-diastolic area index
dIVC_max_: Maximum IVC diameter
dIVC_min_: Minimum IVC diameter
BMI: Body mass index
TEE: Transesophageal echocardiography.
